# Immune Complexes in Juvenile Idiopathic Arthritis

**DOI:** 10.3389/fimmu.2016.00177

**Published:** 2016-05-20

**Authors:** Terry L. Moore

**Affiliations:** ^1^Division of Adult and Pediatric Rheumatology, Saint Louis University Medical Center, SSM Cardinal Glennon Children’s Hospital, St. Louis, MO, USA

**Keywords:** immune complexes, juvenile idiopathic arthritis, rheumatoid factor, anti-CCP antibodies complement levels, affinity chromatography

## Abstract

Juvenile idiopathic arthritis (JIA) reflects a group of clinically heterogeneous, autoimmune disorders in children characterized by chronic arthritis and hallmarked by elevated levels of circulating immune complexes (CICs) and associated complement activation by-products in their sera. Immune complexes (ICs) have been detected in patients’ sera with JIA utilizing a variety of methods, including the anti-human IgM affinity column, C1q solid-phase assay, polyethylene glycol precipitation, Staphylococcal Protein A separation method, anti-C1q/C3 affinity columns, and FcγRIII affinity method. As many as 75% of JIA patients have had IC detected in their sera. The CIC proteome in JIA patients has been examined to elucidate disease-associated proteins that are expressed in active disease. Evaluation of these ICs has shown the presence of multiple peptide fragments by SDS-PAGE and 2-DE. Subsequently, all isotypes of rheumatoid factor (RF), isotypes of anti-cyclic citrullinated peptide (CCP) antibodies, IgG, C1q, C4, C3, and the membrane attack complex (MAC) were detected in these IC. Complement activation and levels of IC correlate with disease activity in JIA, indicating their role in the pathophysiology of the disease. This review will summarize the existing literature and discuss the role of possible protein modification that participates in the generation of the immune response. We will address the possible role of these events in the development of ectopic germinal centers that become the secondary site of plasma cell development in JIA. We will further address possible therapeutic modalities that could be instituted as a result of the information gathered by the presence of ICs in JIA.

Juvenile idiopathic arthritis (JIA) is a protean disorder, whose variable modes of onset and patterns of disease course are accompanied by a myriad of diverse signs, symptoms, and manifestations. It is defined by seven basic subgroups, of which polyarticular-onset rheumatoid factor (RF) positive and negative, oligoarticular-onset, and systemic-onset are the most common onset types (1). Most recent studies show many immune abnormalities characteristic of the disease (2).

## Early Studies

Immune complexes (IC) have been described for many years in patients with JIA. Gabriel and Agnello first found IC in 40% of patients with JIA by the C1q inhibition test and in 24% by the monoclonal RF assay (3). Subsequently, Rossen et al., utilizing the C1q binding assay (C1qBA), reported IC in 22% of children with JIA and their presence correlated with more severe disease (4). Miller et al., using the C1qBA, found IC in 66% with systemic-onset, 38% in polyarticular-onset, but only 14% positive with oligoarticular-onset. This study indicated IC presence correlated with 19S IgM RF and activation products of complement, C3c and C3d in these patients with JIA (5). We, subsequently, assayed 53 children with JIA for IC by four different methods, C1q solid-phase assay (C1qSPA), 2% polyethylene glycol precipitation assay (PEGPA), Raji cell assay (RCA), and the conglutinin assay (KA). Seventy-nine percent of JIA patients demonstrated elevated IC levels by at least one method. By the C1qSPA, 58% were positive, 50% by the RCA, 37% by the KA, but 0% by the PEGPA method. The C1qSPA demonstrated the most elevated levels of IC in all three onset types. The elevated levels correlated with the presence of 19S IgM RF, hidden 19S IgM RF (complexes seen in JIA), and active disease. The correlation with disease activity indicated for the first time that IC may play an active role in the pathophysiology of JIA. The study, also, demonstrated that the C1qSPA was the best assay for measuring IC in the sera of JIA patients (6).

We, subsequently, subjected 10 JIA patients’ sera to affinity chromatography on a rabbit anti-human IgM column eluting with 1 M ammonia and 0.1 M glycine–HCl buffer, pH 3.0, and 15 patients to 4% PEG precipitation followed by acid dissociation of the precipitate. The majority (80%) of the fractions contained IgM RF and IgG, indicating IC. The sera were then subjected to sucrose density gradient (SDG) centrifugation and the resultant fractions analyzed for the presence of IC by the C1qSPA. IC were detected at and ahead of the IgM marker, as expected for IgM RF–IgG complexes (7). A further study isolated IC in JIA patients by the use of immunoabsorbent columns. The IgG fraction obtained by DEAE 50 separation of rabbit anti-human C1q (αHC1q) and goat anti-human C3 (αHC3) antiserum were dialyzed over night against 0.1 M NaHCO_3_ with 0.5 M NaCl, pH 8.3. Finally, the rabbit IgG αHC1q and goat IgG αHC3 were digested with pepsin. The F(ab′)_2_ fragments were isolated by gel filtration on Sephadex G-200 a in 0.15 M PBS pH 7.4 at room temperature. Cyanogen bromide activated Sepharose 4B was swelled and bound to the F(ab′)_2_ fragments of the IgG fractions of αHC1q and αHC3. One milliliter of the patient’s serum was added to the column with 1 ml of 8% PEG in veronal buffer and incubated for 2 h. The mixtures were centrifuged for 1 h. The precipitates were resuspended in 1 ml of veronal buffer and applied to the columns for 60 min at room temperature. The columns were sequentially eluted. The majority again showed IgM RF, IgG, and IgG RF in a smaller percentage. On SDG analysis, all IC demonstrated in the peaks >19S, complement-fixing 19s IgM RF, IgG, and IgG RF containing IC, which proved they can be detected in the serum of JIA patients (8). Furthermore, more sophisticated studies were performed to isolate IC in JIA patients’ sera. JIA sera was evaluated by separation on a Sepharose 4B column to which were bound F(ab′)_2_ fragments of goat IgG anti-human IgM antibody to separate IgM-containing IC. The columns were sequentially eluted and the isolated fractions were assayed for 19S IgM RF and 7S IgG RF by enzyme-linked immunoabsorbent assay (ELISA), IgG levels by immunodiffusion, and by preparative isoelectric focusing (PIEF). The eluates revealed in the ammonia fraction IgM RF in 25% and IgG RF in 16%, all polyarticular-onset JIA patients. In the acid fraction, 67% demonstrated 19S IgM RF and IgG, including both six RF-positive and six RF-negative polyarticular patients and in three oligoarticular patients. IgG RF was also detected in six polyarticular patients. Analysis by PIEF of the IgM RF and IgG RF-positive ammonia and acid fractions showed IgM RF throughout the pH range (4–10), but the highest amount of IgM RF were detected only in the restricted spectrotypic area of pH 4.0–5.5. This was the area where only IgG RF was detected. These studies demonstrated IC containing 19S IgM RF-IgG and 19S IgM RF-7S IgG RF can be detected in JIA sera and predominately found in the pH band 4.0–5.5 by IEF. These complexes that were detected correlated with active disease. This study laid the groundwork for further defining individual constituents of the IC found in JIA patients (9).

Jarvis et al. (10) investigated further the complement system and IC in JIA by examining plasma levels of complement activation fragments C4d and Bb. However, they could find no strong correlations between IC levels and complement activation products. This suggested that plasma complement activation is a concomitant of active disease in JIA (10).

## Later Studies

Subsequent studies were outlined to build on the previous findings to try to determine the possible constituents of these IC. Sera from 104 patients with JIA were separated on a Sepharose 4B column to which were bound F(ab′)_2_ fragments of goat anti-human IgM antibody. The column was sequentially eluted with 1 M ammonia and 0.1 M glycine-HCl buffer, pH 3. The eluted material was treated with sodium dodecyl sulfate (SDS) and simultaneously reduced with 2-mercaptoethanol. Individual components were then separated by SDS-gradient polyacrylamide gel electrophoresis (PAGE) and were transferred to nitrocellulose by Western blotting overlaid with the patient’s own serum and developed with specific antiserum to human IgM and IgG. Four bands were noted in the majority of JIA patients, one in the 70–80 kDa area corresponding to the IgM heavy chain and in the 50 kDa area to the IgG heavy chain. Additional bands specific for JIA were detected in the 40 and 60 kDa areas. Sixty percent of JIA patients showed all four bands and about 80% showed the 40 kDa band and 70% the 60 kDa band. These band findings indicated further the presence of IC in JIA contained IgM RF, IgG RF, and IgG and possibly heat shock proteins (11).

Further studies indicated that circulating IC in JIA have the potential to interact with resident joint synovial fibroblasts (synoviocytes) and induce the expression of inflammatory cytokines. Cultures of normal synoviocytes were treated with IC from the sera of JIA patients that contained IgM RF, C1q, IgG, and C3. These IC induced IL-8 mRNA and protein production. These data further indicate that C1q in these IC mediates IL-8 induction in synoviocytes, playing a key role in the inflammation in the JIA synovium and contributing to disease (12).

Our group subsequently evaluated further IC in JIA patients by attempting to assess B-cell activity by measuring the amount of and the κ:λ immunoglobulin light (L) chain ratio in IC and to determine potential evidence for either an antigen-driven response or B-cell receptor editing. An ELISA was used to measure κ and λ chains present in the IC. The κ:λ light chain ratio was reversed among JIA patients with a λ predominance in RF-positive polyarthritis, RF-negative polyarthritis, oligoarticular patients, and systemic-onset patients. This demonstrated preferential selection of λ chains contributing to the formation of potentially pathogenic IC in JIA patients. This also indicated the potential for L chain editing and a marker for increased B-cell activity in JIA (13).

## Recent Studies

We next evaluated the circulating IC proteome in JIA patients by trying to elucidate disease-associated proteins that were overexpressed in JIA patients with aggressive disease and erosions by X-ray. IC were isolated from the sera of patients with erosive disease or active, early, aggressive disease subsequent to protein separation by 2-DE. Thirty-seven protein spots were overexpressed in the IC from the patients with aggressive disease. These included proteolytic fragments of glyceraldehyde-3-phosphate dehydrogenase, serotransferrin, and α-1-antityrpsin. These disease-associated proteins most definitely contribute to formation of IC and likely have a significant role in disease etiology and pathogenesis. This was the first in-depth analysis of circulating IC in JIA (14). To further evaluate the IC from sera of patients with JIA, we measured activated complement products bound to the circulating IC. Sera from 100 patients with JIA were evaluated for C1q, C4, C3, C3d, and membrane attack complex (MAC) bound to IC by ELISA. JIA patients had elevated levels of complement components bound to their IC, particularly from the classical pathway. This supports the notion of complement-mediated tissue injury that is triggered by IC-mediated classical pathway activation in JIA (15).

Our group subsequently looked at IC constituents through evaluating antibodies to multiple citrullinated epitopes present in the sera of patients with various subtypes of JIA. These studies demonstrated the frequent occurrence of anti-citrullinated type II collagen and anti-citrullinated fibrinogen antibodies (16, 17). Further studies also demonstrated isotypes of α-enolase could also be found in the serum of children with JIA (18).

These studies indicated that citrullinated autoantibody isotype diversity may indicate a more severe disease course in JIA patients. Complement activation and levels of IC overall correlate with disease activity in JIA, indicating their role in the pathophysiology of the disease. These events in protein modification participate in the generation of an immune response and the possible development of ectopic germinal centers that become the secondary site of plasma cell development in JIA. These results indicate that the presence of IC in JIA and especially those containing RF and anti-cyclic citrullinated peptide (CCP) antibody isotypes signify more aggressive disease and earlier intervention with immunomodulators and biologics in the treatment of their disease.

## Author Contributions

The author confirms being the sole contributor of this work and approved it for publication.

## Conflict of Interest Statement

The author declares that the research was conducted in the absence of any commercial or financial relationships that could be construed as a potential conflict of interest.
